# Acute alcohol does not impair attentional inhibition as measured with Stroop interference scores but impairs Stroop performance

**DOI:** 10.1007/s00213-021-05792-0

**Published:** 2021-03-04

**Authors:** P. Riedel, M. Wolff, M. Spreer, J. Petzold, M. H. Plawecki, T. Goschke, U. S. Zimmermann, M. N. Smolka

**Affiliations:** 1 Department of Psychiatry and Psychotherapy, Technische Universität Dresden, Fetscherstraße 74, 01307 Dresden, Germany; 2 Neuroimaging Center, Technische Universität Dresden, Würzburger Straße 35, 01187 Dresden, Germany; 3 UCLA Semel Institute for Neuroscience & Human Behavior, David Geffen School of Medicine, 760 Westwood Plaza, Los Angeles, CA 90024 USA; 4 Department of General Psychology, Technische Universität Dresden, Zellescher Weg 17, 01069 Dresden, Germany; 5 Department of Psychiatry, Indiana University School of Medicine, 355 West 16th Street, Indianapolis, IN 46202 USA; 6 Department of Addiction Medicine and Psychotherapy, kbo-Isar-Amper-Klinikum München-Ost, Vockestraße 72, 85540 Haar, Germany

**Keywords:** Response inhibition, Interference control, Acute alcohol exposure, Alcohol clamp method, Stroop task

## Abstract

**Rationale:**

Inhibition is a core executive function and refers to the ability to deliberately suppress attention, behavior, thoughts, and/or emotions and instead act in a specific manner. While acute alcohol exposure has been shown to impair response inhibition in the stop-signal and Go/NoGo tasks, reported alcohol effects on attentional inhibition in the Stroop task are inconsistent. Notably, studies have operationalized attentional inhibition variably and there has been intra- and inter-individual variability in alcohol exposure.

**Objective:**

This study aimed to examine the acute effects of alcohol on attentional inhibition, considering previous limitations.

**Methods:**

In a single-blind, cross-over design, 40 non-dependent participants with a medium-to-high risk drinking behavior performed a Counting Stroop task (CST) under a baseline and an arterial blood alcohol concentration (aBAC) clamp at 80 mg%. Attentional inhibition was assessed as the alteration of reaction times (RT), error rates (ER), and inverse efficiency scores (IES) between incongruent and congruent trials (interference score). Stroop performance was also assessed regardless of trial-type.

**Results:**

Compared to saline, acute alcohol exposure via an aBAC clamp did not affect CST interference scores but increased RTs and IES in both incongruent and congruent trials.

**Conclusions:**

Attentional inhibition (Stroop interference score) was not impaired by clamped moderate alcohol exposure. Acute alcohol impaired Stroop performance evidenced by a general increase in response times. Our findings suggest that response and attentional inhibition do not share the same neurocognitive mechanisms and are affected differently by alcohol. Results could also be explained by automated behaviors known to be relatively unaffected by acute alcohol.

**Supplementary Information:**

The online version contains supplementary material available at 10.1007/s00213-021-05792-0.

## Introduction

In experimental psychology, inhibition is considered a core executive function (Miyake et al. 2000; Miyake and Friedman 2012) and refers to the ability to deliberately suppress or override prepotent attention, behavior, thoughts and/or emotions and instead act on what is currently more appropriate (Diamond 2013). Inhibition thus involves a number of different facets (Diamond 2013; Bender et al. 2016), two of which are response inhibition (or behavioral inhibition) and attentional inhibition (or interference control) (Diamond 2013; Bender et al. 2016; Tiego et al. 2018). Response inhibition requires the suppression of a prepotent motor response. Attentional inhibition requires suppressing attention towards distracting (i.e., interfering) stimuli.

Research demonstrated that acute alcohol exposure impairs response inhibition (e.g., Marczinski, Abroms, Van Selst, & Fillmore, 2005). Response inhibition has been studied with a high mean arterial Blood Alcohol Concentration (aBAC) of more than 100 mg% (Stock et al. 2016) or using an alcohol clamp to assure aBACs at 60 mg% in each individual of a participant sample (Gan et al. 2014). In contrast, acute effects of alcohol on attentional inhibition are less clear. Alcohol has not been shown to acutely effect attentional inhibition as measured with Stroop interference scores, that is, the performance difference in incongruent compared to congruent trials (Marinkovic et al. 2012; Bartholow et al. 2018; details below). Alcohol effects are inconsistent when attentional inhibition is examined using total Stroop performance (i.e., total time to complete a set of incongruent trials) instead of interference scores (Duka and Townshend 2004; Rose and Duka 2007, 2008). In addition, previously used versions of the Stroop task did not include a natural mapping between stimulus and response and therefore may have introduced variability in performance unrelated to attentional inhibition. Furthermore, past studies used only mean aBAC levels of up to 70 mg% and no aBAC clamp. Consequently, the current study aimed to examine Stroop interference scores as well as total performance using a Counting Stroop task and a moderate aBAC clamp at 80 mg%.

Impairments in response inhibition and attentional inhibition are captured using different tasks (Wolff et al. 2016; Tiego et al. 2018). Specific aspects of response inhibition can be measured with the stop-signal task (SST) (Logan et al. 1984; Verbruggen and Logan 2008) and the Go/NoGo task (Wolff et al. 2016). The SST requires the suppression of a triggered and already initiated motor response by presenting an inhibitory signal after the Go stimulus (Meyer and Bucci 2016) and therefore measures reactive response control. The Go/NoGo task requires the suppression of a common motor response that is not yet initiated by presenting a no-go signal instead of the stimulus (Littman and Takács 2017) and therefore measures proactive response control. Research systematically demonstrated that acute alcohol exposure impairs inhibition in the SST (de Wit et al. 2000; Loeber and Duka 2009; Gan et al. 2014; Roberts et al. 2016; Yan et al. 2016) and the Go/NoGo task (Fillmore and Weafer 2004a; Marczinski et al. 2005; Field et al. 2010; Korucuoglu et al. 2015; Stock et al. 2016). The acute alcohol effect on response inhibition was observed in participants with both binge drinking (Stock et al. 2016) and social drinking patterns (Roberts et al. 2016).

Attentional inhibition can be captured using the Stroop task (Stroop 1935; MacLeod 1991, 2014), the Eriksen flanker task (Eriksen and Eriksen 1974), the Simon task (Simon 1969), and oculomotor response tasks (Abroms et al. 2006). Oculomotor response tasks measure the ability to inhibit eye movement (saccades) to distractor stimuli. In a Simon task, participants are asked for a spatial response (left or right button press) corresponding to two different non-spatial stimulus features (e.g., two colors) and to inhibit a prepotent spatial response. In an Eriksen flanker task, participants are instructed to attend to a target stimulus and suppress attention towards task-irrelevant flankers. In the Stroop task, a stimulus conflict requires the suppression of attention towards a prepotent but task-irrelevant stimulus, and the response to a task-relevant stimulus. There is an ongoing debate whether these tasks measure a common construct (i.e., form one latent factor) (Rey-Mermet et al. 2018; Tiego et al. 2018; Paap et al. 2020). However, there is agreement that the tasks (and/or adaptations of the tasks) measure different specific features and that no general conclusions regarding attentional inhibition can be drawn from a single task and vice versa (Donohue et al. 2016; Scerrati et al. 2017; Bartholow et al. 2018; Hübner and Töbel 2019).

Attentional inhibition tasks differ in their susceptibility to acute alcohol exposure. Results for the flanker task indicate that, given sufficient training and the presence of a high proportion of conflict trials, there is no impairment of attentional inhibition by acute alcohol exposure (Bartholow et al. 2003). However, acute alcohol exposure specifically impairs the capacity to enhance attentional inhibition following error trials (Bartholow et al. 2012; Bailey et al. 2014). Acute alcohol intake did not affect accuracy and reaction times (RTs) of both a simple Simon task and a hybrid Simon-Stroop task (Rosen et al. 2016). With regard to oculomotor tasks, results indicate that intentional aspects of attentional inhibition are clearly susceptible to acute alcohol exposure and automatic aspects are not (Abroms et al. 2006).

The present study concentrated on attentional inhibition as measured with Stroop interference and total scores. Most studies on the effects of acute alcohol exposure used the color-word version of the Stroop task. In the Color Stroop task, participants are presented with color words in incongruent color (e.g., RED depicted in blue color) and asked to report the color of the word and resist to read the word (prepotent response). Here it is assumed that the greater the total time to complete the task (i.e., latency) or the higher the total error rate (ER), the poorer the inhibition. In more recent studies, congruent trials (e.g., RED depicted in red color) are presented in addition to incongruent trials. Here it is assumed that the greater the RTs or the higher the ER in the incongruent trials compared to the congruent trials (i.e., interference scores), the poorer the inhibition.

There are a number of studies that used only incongruent stimuli to examine the effects of acute alcohol exposure on attentional inhibition. Duka and Townshend (2004) did not find an impact of low levels of acute alcohol exposure (aBAC of about 15 to 40 mg%) on latency and total ER in a Color Stroop task. Notably, they did find an acute alcohol exposure associated increase in ER when using alcohol-related stimuli instead of color-word stimuli. However, the authors noted that alcohol-related stimuli may have activated “mental representations of alcohol-related behaviours” (Duka and Townshend 2004), and therefore assumed that the results could be explained by effects of low alcohol exposure levels on these cognitive representations rather than the effects of alcohol on inhibition. Rose and Duka (2007, 2008) later found increased ERs under moderate alcohol exposure levels (estimated peak aBAC of about 70 mg%) for both color-word and alcohol-related stimuli.

In two separate studies using only incongruent color-word stimuli, Gustafson and Kallmen (1990a, 1990b) found an increase in latency under moderate alcohol exposure levels (group mean aBAC of about 60 to 80 mg%) compared to placebo (non-alcoholic drink with alcohol essence). However, they did not find such an effect when compared to a control group (non-alcoholic drink without alcohol essence). Because the placebo group showed lower latency in comparison to both the alcohol group and the control group, the authors interpreted their results as a “compensatory effort” during placebo rather than an acute alcohol effect on inhibition. Another study indicated that higher latency in an alcohol condition might also be explained by high motivation, which results in a speed–accuracy trade-off in favor of lower ERs (Gustafson and Kallmen 1990c).

Taken together, the results of studies using only incongruent stimuli are mixed. In addition, these studies cannot entirely exclude the possibility that the reported effects of acute alcohol exposure on inhibition are secondary to a general response slowing. Therefore, studies using both incongruent and congruent stimuli and interference scores (Marinkovic et al. 2012; Bartholow et al. 2018) may be more informative. In line with studies using only incongruent stimuli, Bartholow et al. (2018) recently showed an increased ER for incongruent trials under moderate but variable alcohol exposure levels (group mean aBAC of about 50 to 70 mg%). Importantly, Bartholow et al. found no effect of acute alcohol exposure on interference scores. Similarly, Marinkovic et al. (2012) found increased RTs under a group mean aBAC of about 40 to 50 mg%, but no effect on interference scores. Although these results suggest no effect of moderate alcohol levels on attentional inhibition, variability in aBAC levels within and between participants in these studies is greater than could be achieved with an aBAC clamp (Ramchandani et al. 1999, 2009; O’Connor et al. 2000). It may even be the case that some individuals do not reach moderate aBAC levels after oral alcohol intake and, if testing is matched to the trajectory of exposure, potential limb or rate effects are introduced (Pohorecky 1977; Martin and Earleywine 1990; King et al. 2002; Pihl et al. 2003; Morris et al. 2017; Bartholow et al. 2018).

The current study addresses a gap in the existing literature in several ways. First, we examined the acute alcohol exposure effects on attentional inhibition using Stroop interference scores (i.e., difference in responses to incongruent compared to congruent trials) in addition to the total RT, ER, and inverse efficiency score (IES). Second, the Counting Stroop task (CST) was used instead of the Color Stroop task. The major benefit of the CST is the natural mapping between stimulus and response, that is, the correct response is further to the right for higher presented digit value. As a result, the impact of alcohol exposure on cognitive domains, such as working memory and learning ability, were minimized. Third, to further reduce intra- and inter-individual variability in aBACs, we administered alcohol through an intravenous infusion procedure (alcohol clamp method; O’Connor et al. 1998; Gan et al. 2014). Participants were asked to perform the CST with a moderate aBAC clamp of 80 mg% compared to a placebo session (intravenous infusion of normal saline). The assessment was part of a larger clinical research project and the sample was specifically selected for medium-to-high risk drinking behavior.

## Materials and methods

This study was part of a larger clinical research project (https://clinicaltrials.gov/ct2/show/NCT02652585, EudraCT Number: 2015-002831-16, Sponsor Protocol Number: TUD-TEMANX-065). All study procedures were approved by the Koordinierungszentrum für Klinische Studien Dresden (KKS-DD; Coordination Center for Clinical Studies Dresden) and the Ethics Committee of the Technische Universität Dresden (TUD). Written informed consent was obtained from each participant. All participants were unaware of the hypotheses of the study. Participants received approximately €300.00 as compensation for participating in this study.

Participants were randomized at the beginning of the clinical trial to either receive the nonselective opioid receptor antagonist naltrexone or placebo in a randomized, double-blind design over the entire course of the study (see supplement for details). The Counting Stroop task (CST) was a secondary outcome measure of the clinical trial and is the focus of the study at hand. CST data was collected from 18 February 2016 to 31 August 2017 at the Neuroimaging Center of the TUD.

### Procedure

The CST was performed twice: first on clinical study visit 3 (from here on referred to as the first session) and then on clinical study visit 4 (now referred to as the second session). At the beginning of each session, participants underwent laboratory screening, an assessment of vital signs, and surveys (alcohol use, withdrawal symptoms, adverse events, potential side effects of the study medication since the last clinical study visit). Subsequently, intravenous infusion of alcohol or placebo (normal saline) was randomly administered in a single-blind, cross-over design (see “Alcohol administration” below). Approximately 25 min after the start of the infusion, during which either an aBAC clamp of 80 mg% (alcohol condition) or an aBAC of 0 mg% (placebo condition) was performed, participants started the CST. The CST was completed in about 8 min and at a clamped alcohol exposure or under placebo (see “Counting Stroop task” below). No other tasks were performed prior to or after the CST. After the CST, participants underwent neuroimaging (Fang et al., under review). Subjective alcohol effects were indicated on a visual analogue scale (I) before the intravenous infusion, (II) before the CST, and (III) after neuroimaging (see supplement).

### Participants

Forty-six participants consented and were eligible for the study. Six participants did not complete two CST sessions (no CST session: *N* = 4; one CST session: *N* = 2). Forty women and men from age 25 to 55 were included in the study (Mean (*M*) = 29.4, standard deviation (SD) = 4.7). All participants were white and showed proficiency in the German language. Participants were right-handed as assessed with the Edinburgh Handedness Inventory (Oldfield 1971) and reported normal or corrected-to-normal vision.

Alcohol consumption of each participant, as assessed with the Timeline Followback Interview (Sobell and Sobell 1992), met the following criteria within the last 45 days: (a) alcohol was consumed at least once a week at medium risk (World Health Organization (WHO) 2000; European Medicines Agency (EMA) 2010), (b) average alcohol consumption was at least 41 g/day for men and 31 g/day for women, (c) there were at least 6 days with an alcohol consumption of >100 g/day for men or 75 g/day for women, (d) there were at least 4 consecutive alcohol abstinence days. Importantly, a current or previous alcohol or other substance use disorder (except nicotine) was excluded in all participants using the World Health Organization World Mental Health Composite International Diagnostic Interview (WHO WMH-CIDI; WHO, 1990). Participants had no past treatment related to alcohol consumption (including counseling and support groups). Furthermore, they had no history of alcohol withdrawal, seizures or delirium. Additionally, participants were screened for exclusion criteria for alcohol administration (e.g., pancreatitis, cirrhosis of the liver, hypersensitivity to alcohol). Individuals were excluded if pregnant (determined via urine pregnancy test) or breastfeeding. Intake of alcohol or illicit drugs was ruled out at each session using established screening methods (interview, alcohol breath test, urine screening). In summary, all participants had a medium-to-high risk drinking behavior, but not alcohol use disorder and were fit to abstain from alcohol without developing withdrawal symptoms. The sample was therefore appropriate to meet the objectives of the clinical trial.

Apart from excluding individuals with substance use disorders, individuals with other neuropsychiatric disorders requiring treatment were also excluded from participation. For this purpose we used the Mini-International Neuropsychiatric Interview (MINI; Sheehan et al., 1998). Participants had no history of suicide attempts and did not currently take psychotropic medication or opioid analgesics. Given the larger scope of the clinical trial, participants were also screened for exclusion criteria related to (a) magnetic resonance imaging (MRI) and (b) naltrexone administration (according to the national expert information). Clinical trial participation in the past 4 weeks was also an exclusion criterion. In each session, participants underwent a medical interview carried out by a physician that included the Clinical Institute Withdrawal Assessment for Alcohol (CIWA) (Stuppaeck et al. 1994), the Beck Depression Inventory (BDI-II) (Beck et al. 1996), and a monitoring of adverse events/side effects and life events since the last visit. Participants also underwent a medical screening (e.g., vital signs) in each session.

### Counting Stroop task

**Fig. 1:**
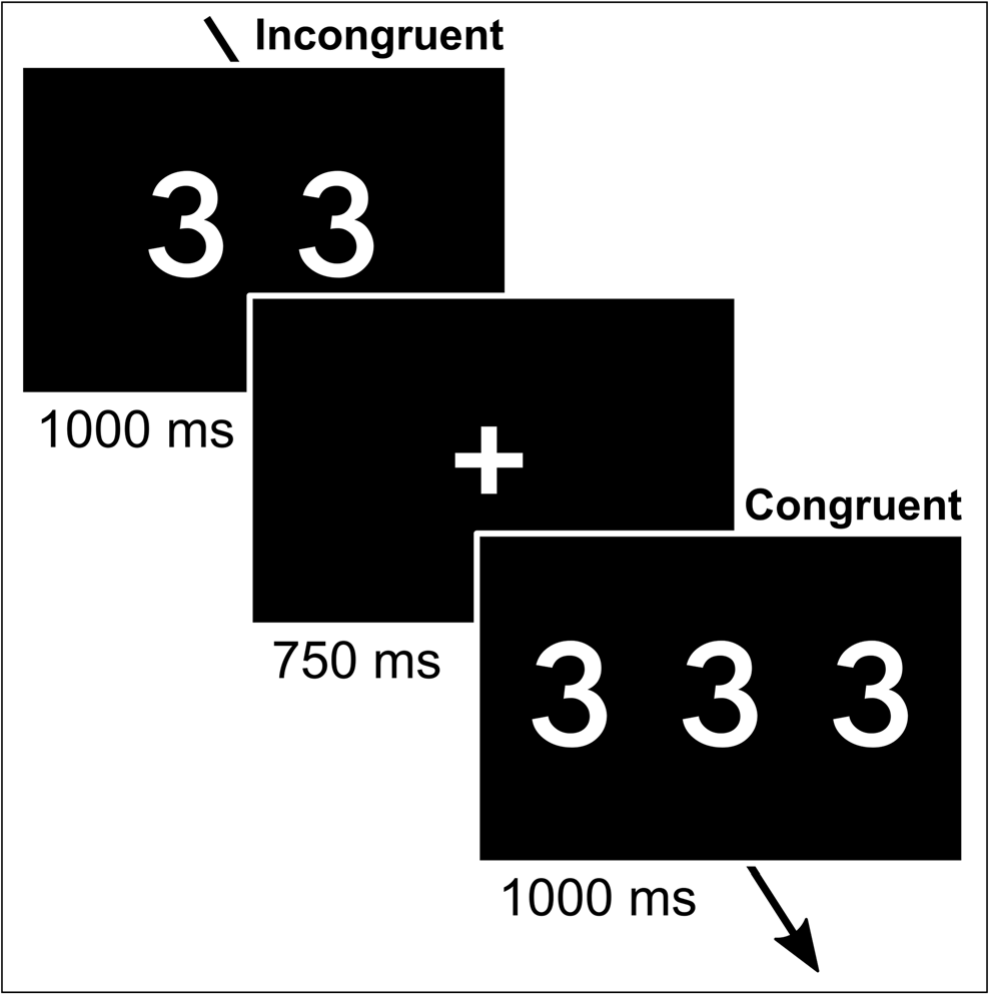
Task design. The Counting Stroop task (CST) was implemented as described by Wolff et al. (2016) (see Methods). The figure shows an example of an incongruent trial (left) and a congruent trial (right). Participants were asked to ignore denotations, but to respond according to the amount of presented digits. There were four response keys that were naturally mapped from left to right on the keyboard (QWERTZ layout) in increasing numbers (“*Y*” = 1, “*C*” = 2, “*B*” = 3, and “*M*” = 4)

The CST comprised 160 trials with no inter-trial interval and without feedback as described by Wolff et al. (2016) (Fig. 1). Each trial consisted of a fixation cross (750 ms; fixed) and a subsequent stimulus (1000 ms). The stimulus consisted of the presentation of one, two, three, or four identical digits from 1 to 4. The task included 80 incongruent and 80 congruent trials (within-subject factor trial-type (Incongruent/Congruent)). In congruent trials, the number of presented digits matched the denotation. For example, the digit “3” (denotation) was presented three times (i.e., 333). In incongruent trials, the number of presented digits did not match the denotation. For example, the digit “3” (denotation) was presented two times (i.e., 33) (Fig. 1). Participants were instructed to ignore denotations (i.e., to resist prepotent response), but to respond according to the number of presented digits. There were four response keys that were naturally mapped from left to right on the keyboard (QWERTZ layout) in increasing numbers (“*Y*” = 1, “*C*” = 2, “*B*” = 3, and “*M*” = 4). The correct response, for example for “2222” was “*M*,” which is the fourth response button. Trial-types were presented in pseudorandom order.

The CST was conducted in both sessions (within-subject factor session (First/Second)) under either an aBAC clamp of 80 mg% or under an aBAC of 0 mg% (within-subject factor alcohol (Alcohol/NaCl); see below). Prior to the task, and before the aBAC clamp was established, participants received instructions for the task and completed a short training exercise. Task presentation was performed using Matlab 2017a (The MathWorks Inc., Natick, Massachusetts, USA).

### Alcohol administration

At the beginning of the clinical trial, the alcohol and placebo session order was randomized. One group of participants received an intravenous infusion of alcohol (6% v/v; mixture of normal saline with 95% ethanol [Braun, Melsungen, Germany]) during the first session and an intravenous infusion of normal saline (sodium chloride [NaCl] 0.9% solution) during the second session (*N* = 18) while in the other group, the order was reversed (*N* = 22). This resulted in the between-subjects factor order (Alcohol_1st_Session/Alcohol_2nd_Session), which was counterbalanced across the sample. Participants were instructed that a higher amount of alcohol would be administered in one session and a lower amount in the other, with a maximum aBAC of 80 mg%. Details on blinding and on the assessment of subjective alcohol effects are reported in the supplement. After the experiment, participants were fully debriefed.

Participants presented with an aBAC of 0 mg% at each appointment. Alcohol administration was performed as in previous studies, that is, via an alcohol clamp method (O’Connor et al. 1998; Gan et al. 2014) and continuously monitored by a physician. Based on height, weight, age and sex, infusion rate protocols were calculated using the CAIS software to bring the aBAC from 0 to 80 mg% within 25 min and then maintain it at this level for another 60 min (CAIS software; proprietary software developed by the Indiana Alcohol Research Center, Indiana University School of Medicine, Indianapolis, IN, USA, mplaweck@iupui.edu). Repeated aBAC readings were obtained during each infusion (*N* = 1 as baseline and *N* = 5 during infusion) and entered into the CAIS software in real time to adapt the prescribed infusion rates to control the resultant alcohol exposure.

### Alcohol concentration readings

aBAC readings were obtained using an Alcotest 6810 med breathalyzer (Dräger Sicherheitstechnik, Lübeck, Germany). The breathalyzer measured alcohol concentration in end-expiratory breath, which is closely related to aBAC during intravenous ethanol infusion (Lindberg et al. 2007). As alcohol exposure is conventionally communicated as BAC, the breathalyzer applied the usual 1:2100 air/blood partition coefficient to approximate aBAC (mg%) from breath readings (mg of ethanol/liter of air). Due to the high cerebral perfusion index, aBAC provides a reliable estimate of brain alcohol exposure, which is the key factor driving both behavior and subjective alcohol effects. Participants were kept blind to the aBAC readings as there is a short delay between exhalation and the determination as well as display of the measurement.

### Calculation of outcome measures

Outcome measures were calculated as described by Wolff et al. (2016) and consisted of the RTs, ERs, and IES on incongruent and congruent trials as well as their interference scores (incongruent-congruent trials) for each session. Mean RTs and ERs were calculated for each participant per trial-type (Incongruent/Congruent) and session (First/Second and Alcohol/NaCl, respectively). Misses were regarded as error trials. RTs for error trials were not included in the statistical analysis. Within each participant, RTs that deviated from the participant’s median in the respective condition (Incongruent/Congruent) and session by more than 3.32 median absolute deviations were excluded (Wilcox and Keselman 2003; Friedman et al. 2008).

Interference scores were calculated by subtracting RT, ER, and IES scores in congruent trials from incongruent trials. Calculation of outcome measures was performed using Matlab 2017a (The MathWorks Inc., Natick, MA, USA).

CST performance could not be appropriately quantified by outcome measures based on either RTs or ERs alone as the speed–accuracy trade-off may be balanced differently by individuals (Heitz 2014; Bogacz 2015). Therefore, RTs and ERs were additionally combined into inverse efficiency scores (IES = RT/[1-ER]) (Townsend and Ashby 1983; Bruyer and Brysbaert 2011).

To reduce the influence of extreme scores and to improve normality, participant’s overall RT, ER, and IES scores that deviated from the group mean by more than three SDs were replaced with values exactly three SDs from the mean. Participant’s outcome measures were excluded from statistical analyses when accuracy was below chance. That is, when the binomial probability that a participant would have obtained a higher-than-observed accuracy by chance was > 0.01 (Friedman et al. 2008). More specifically, for the CST (chance hit probability of 25 %), this approach resulted in the exclusion of participants with an accuracy below about 35 % in at least one trial-type. This procedure ensured that only participants that had understood and followed task instructions were eligible for statistical analysis.

### Statistical analysis

Subsequent statistical analyses were performed in R version 3.4.3 (R Core Team 2017). For all statistical tests, the level of significance was defined at 5 % (*α* = 0.05). In order to focus statistical power on the effects of acute alcohol exposure on RT, ER, and IES interference and total scores, we only used the within-subject factors trial-type (Incongruent/Congruent) and alcohol (Alcohol/NaCl) for the main analyses. The between-subjects factor order (Alcohol_1st_Session/Alcohol_2nd_Session) was dropped after supplemental analyses revealed no influence of order on expected session/training effects (see supplement).

Next, we used G*Power (Faul et al. 2007) to compute down to what effect size (Cohen’s *d*) an influence of acute alcohol exposure on attentional inhibition could be detected in our sample (*N* = 40). We based these calculations on a paired sample two-tailed t-test on the RT interference scores (mean RT incongruent trials–mean RT congruent trials). The paired t-test is equivalent to the 2 × 2 factorial analysis of variance (ANOVA) that was used for our main analyses (see below). Reliability of the CST was assessed as in previous studies (Wolff et al. 2016, 2020). That is, internal consistency (Cronbach’s *α*) was calculated by adjusting split-half correlations with the Spearman-Brown prophecy formula (Brown 1910).

Next, we tested the effect of acute alcohol on Stroop performance. A 2 × 2 factorial repeated measures ANOVA with the within-subject factors trial-type (Incongruent/Congruent) and alcohol (Alcohol/NaCl) was performed using the R package afex version 0.26. Repeated measures ANOVAs were separately performed for RTs, ERs, and IES. For significant effects, numerical differences in estimated marginal means were computed using the R package emmeans version 1.4.4.

In addition to the F-statistic, we computed the Bayes statistics for our ANOVA model using the R package BayesFactor version 4.2 and the anovaBF function (see details in Rouder et al., 2012). In contrast to the frequentist approach, the Bayesian approach “allows us not only to provide evidence against the null hypothesis but also in favor of it” (Kass and Raftery 1995; Ortega and Navarrete 2017). The Bayes factor demonstrates how much more probable one model is against another model, with no special status assigned to the null hypothesis. We compared the full and all reduced models against a denominator that included neither main nor interaction effects of alcohol and trial-type. In addition, we directly assessed whether and to what extent the Bayes factor preferred the model that included only the main effects of alcohol and trial-type against the model that additionally included the alcohol × trial-type interaction.

Three additional supplemental analyses were performed to see if any alcohol × trial-type interaction was masked, that is, whether it appeared only in a specific property of the task. First, we used Deltaplots (Ridderinkhof et al. 2005; Burle et al. 2014) to examine potential effects specific to RT quantiles. We performed a 2 × 4 repeated measures ANOVA with the within-subject factors alcohol and RT quantile (1/2/3/4) on the dependent variable RT interference score (mean RT incongruent trials–mean RT congruent trials). Second, we assessed whether statistical effects were affected by the type of response (button). We performed a 2 × 4 repeated measures ANOVA with the within-subject factors alcohol and button press (“*Y*” = 1, “*C*” = 2, “*B*” = 3, and “*M*” = 4) on the dependent variable RT interference score. Third, we performed a 2 × 2 × 2 factorial repeated measures ANOVA with the within-subject factors alcohol, current trial-type and previous trial-type on the dependent variable RT to assess potential effects of alcohol on conflict adaptation, that is, the Gratton effect (Gratton et al. 1992).

Neither naltrexone (van Steenbergen et al. 2017) nor other opioid receptor antagonists (Chamberlain et al. 2012) have been shown to alter interference scores in the Stroop task. Hence, this factor was not included in the current analyses. Analyses on the subsample of participants that were assigned to the placebo arm are presented in the supplement. Results of the naltrexone intervention, neuroimaging and assessments performed at the other clinical study visits are reported elsewhere (e.g., Fang et al., under review; Spreer et al., in preparation).

## Results

Forty participants were included in the statistical analysis of the behavioral data reported in this study. Basic characteristics of the sample are presented in Table 1. Descriptive data on subjective alcohol effects are reported in the supplement (Fig. S1). Descriptive statistics for RTs, ERs, and IES are also provided in the supplement (Tables S2–4). Given the sample size and design of the study, an effect of acute alcohol exposure on interference scores could have been detected down to a Cohen’s *d* of 0.58. Internal consistency of the CST was good for both the alcohol (Cronbach’s *α*: RT = 0.89 ER = 0.59 IES = 0.83) and the NaCl condition (Cronbach’s *α*: RT = 0.83, ER = 0.60, IES = 0.83), and comparable to the internal consistency of the same task in a previous study (Cronbach’s *α* = 0.60 in Wolff et al., 2016).

**Table 1:** Basic characteristics of the participant sample. All participants had a medium-to-high risk drinking behavior as assessed with the Timeline Followback Interview. Current or previous alcohol use disorders were excluded in all participants

***N*** **total**	40
**Age (mean ± standard deviation)**	29.4 ± 4.7
***N*** **females**	4
**Higher education**	
*N* completed college/university/apprenticeship training	23
*N* current college/university/apprenticeship training	14
*N* no college/university/apprenticeship training	3
***N*** **high school or high school equivalent**	40
***N*** **daily nicotine intake**	27
**Timeline Followback (past 45 days)**	
Drinking days (%)	70
Binge drinking days (%)	47
Alcohol intake (g) on drinking days (mean ± standard deviation)	114 ± 41.4

**Fig. 2:**
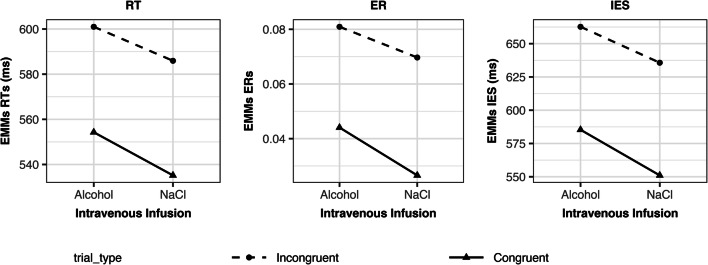
Effects of alcohol and trial-type on reaction times (RT; left), error rates (ER; middle), and inverse efficiency scores (IES; right). Estimated marginal means (EMM) of RTs, ERs, and IESs for each trial-type are shown as a function of alcohol. For an overview of the results of the statistical analyses, please refer to Table 2

Statistical results of the main analysis on the CST are presented in Table 2 and Fig. 2. Equivalent results were obtained for RTs, ERs, and IES (see Table 2). ERs were generally low (*M* = 0.055, SD = 0.052). Therefore, we focus only on RTs from here on. A 2 × 2 factorial repeated measures ANOVA yielded no significant alcohol × trial-type interaction (F(1,39) = 0.94, *p* = 0.337, η^2^_G_ < 0.001). There were significant main effects of alcohol (F(1,39) = 8.45, *p* = 0.006, η^2^_G_ = 0.02) and trial-type (F(1,39) = 208.73, *p* < 0.001, η^2^_G_ = 0.16). That is, alcohol increased RTs by 17 ms (EMM) regardless of trial-type. RTs were increased by 49 ms (EMM) for incongruent compared to congruent trials.

**Table 2:** F-statistic: main and interaction effects of 2 × 2 factorial repeated measures ANOVA for reaction times (RT; left), error rates (ER; middle), and inverse efficiency scores (IES; right). Values rounded to two decimals. *DFn* degrees of freedomin the numerator, *DFd* degrees of freedom in the denominator, * significant, *η*^*2*^_*G*_ generalized eta-squared

Effects	DFn	DFd		RT				ER				IES		
			*F*	*p*		η^2^_G_	*F*	*p*		η^2^_G_	*F*	*p*		η^2^_G_
(Intercept)	1	39	5022.11	< 0.001	*	0.99	44.44	< 0.001	*	0.38	2867.06	< 0.001	*	0.98
Alcohol	1	39	8.45	0.01	*	0.02	1.24	0.27		0.01	4.69	0.04	*	0.03
Trial-type	1	39	208.73	< 0.001	*	0.16	35.83	< 0.001	*	0.07	95.49	< 0.001	*	0.17
Alcohol × trial-type	1	39	0.94	0.34		< 0.01	0.30	0.59		< 0.01	0.39	0.54		< 0.01

In addition to the F-statistic, we used Bayes factor analysis to compare models. The preferred model included main effects of alcohol and trial-type but no interaction (Table 3). That is, an acute effect of alcohol on the total RT and IES, regardless of a separate effect of trial-type, was most likely. When directly comparing the full model including the alcohol × trial-type interaction and both main effects against the reduced model including both main effects but no interaction, the Bayes factor was 0.29. Hence, the Bayes factor in favor of the reduced model assuming no effect of alcohol on interference scores was 1/0.29 = 3.5. A Bayes factor of 3.5 indicates moderate evidence (Jeffreys 1961; Lee and Wagenmakers 2013) of no effect of acute alcohol exposure on interference scores, but only on absolute RT and IES.

**Table 3:** Bayes-statistic: Bayes factors for full and reduced models separately for reaction times (RT; top), error rates (ER; middle), and inverse efficiency scores (IES; bottom). For each measure, all models were first compared against a denominator assuming no effects. Subsequently, the full model including main and interactions effects was compared against the denominator assuming main effects only. A proportional error estimate is presented next to the Bayes factor. Values rounded to two decimals

**RT**	**Model** (against denominator: no effects)	**Bayes factor**	
	Main effect trial-type + main effect alcohol	3.54E+19	±3.21%
	Main effect trial-type + main effect alcohol + interaction effect	1.02E+19	±9.65%
	Main effect trial-type	1.15E+17	±12.39%
	Main effect alcohol	6.01	±0.89%
	**Model** (against denominator: main effect trial-type + main effect alcohol)	**Bayes factor**	
	Main effect trial-type + main effect alcohol + interaction effect	0.29	±10.17%
**ER**	**Model** (against denominator: no effects)	**Bayes factor**	
	Main effect trial-type + main effect alcohol	494.14	±2.88%
	Main effect trial-type + main effect alcohol + interaction effect	119.89	±2.37%
	Main effect trial-type	928.02	±3.07%
	Main effect alcohol	0.47	±0.99%
	**Model** (against denominator: main effect trial-type + main effect alcohol)	**Bayes factor**	
	Main effect trial-type + main effect alcohol + interaction effect	0.24	±3.73%
**IES**	**Model** (against denominator: no effects)	**Bayes factor**	
	Main effect trial-type + main effect alcohol	3.23E+10	±2.35%
	Main effect trial-type + main effect alcohol + interaction effect	7.34E+09	±1.49%
	Main effect trial-type	2.88E+09	±1%
	Main effect alcohol	2.7	±1.94%
	**Model** (against denominator: main effect trial-type + main effect alcohol)	**Bayes factor**	
	Main effect trial-type + main effect alcohol + interaction effect	0.23	±2.79%

**Fig. 3:**
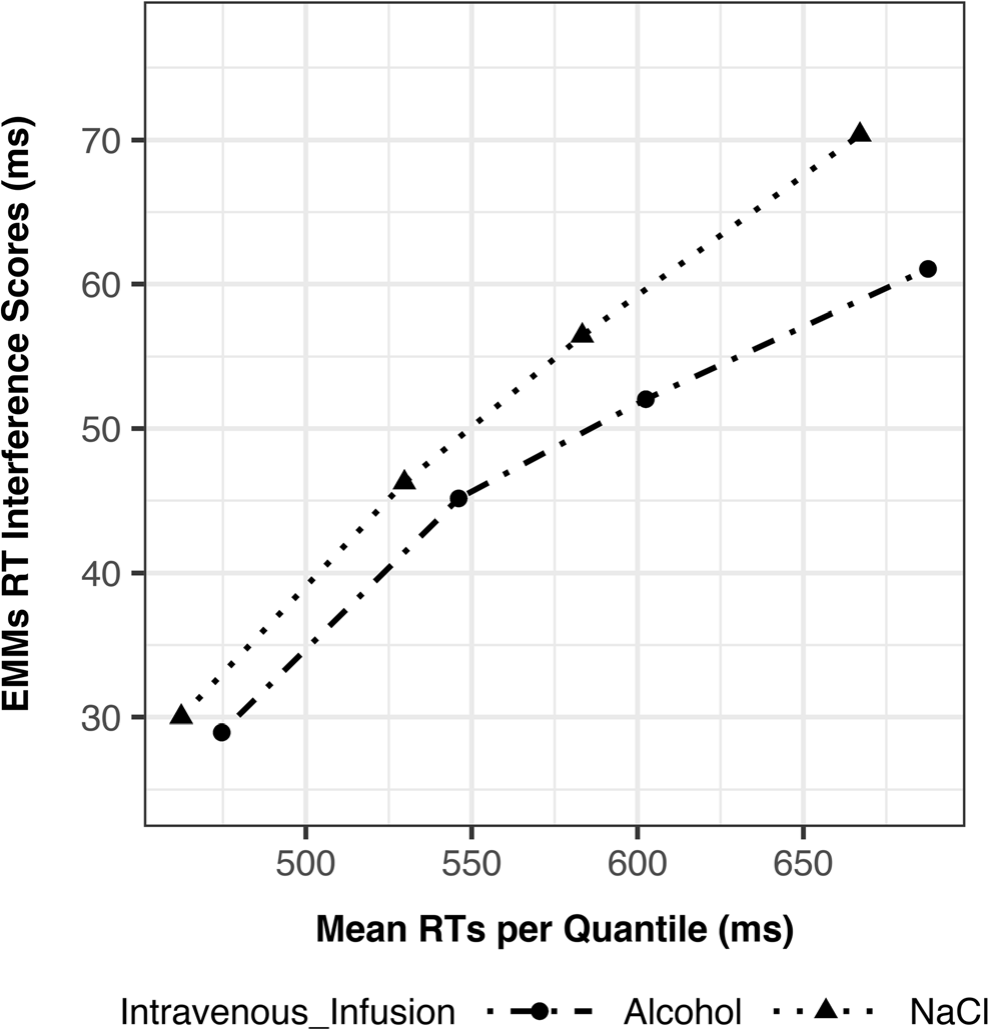
Deltaplot of interference scores by reaction time (RT) quantiles and alcohol. Estimated marginal means (EMM) of RT interference scores are shown as a function of alcohol across four RT-quantiles. RT interference scores are defined as the RT difference between incongruent and congruent trials

Supplemental analyses examined more specific alcohol exposure effects on attentional inhibition: effects on interference scores at certain RT quantiles and effects on conflict adaptation. Using Deltaplots, the effects of alcohol on the RT interference score are presented for different RT quantiles (Fig. 3). A 2 × 4 factorial repeated measures ANOVA showed a significant main effect of RT quantile (F(3,117) = 52.48, *p* < 0.001), that is, interference scores increased between quantile 1 to 4 (Fig. 3). However, there was no significant alcohol × RT quantile interaction (F(3,117) = 0.96, *p* = 0.413), that is, there was no effect of alcohol on the RT interference score that was specific to any RT quantile.

Further analyses revealed significant conflict adaptation (i.e., Gratton effect) (F(1,39) = 47.83, *p* < 0.001), that is, a reduced interference score after incongruent trials compared to after congruent trials. However, alcohol did not affect conflict adaptation (F(1,39) = 0.10, *p* = 0.754) (for details please refer to the supplement). In addition to these analyses, we excluded any effect of alcohol on the RT interference score attributable to any of the four response buttons (F(3,114) = 0.22, *p* = 0.880) (for details please refer to the supplement).

## Discussion

Our analyses showed three main findings. The CST proved effective in measuring attentional inhibition at a clamped aBAC of 80 mg%. Attentional inhibition, as measured with interference scores in the CST, was not acutely affected by this moderate and constant alcohol exposure. Acute alcohol exposure increased RTs and IES in the CST regardless of trial-type.

The CST yielded several expected effects of session and trial-type. RTs, ERs and IES increased in incongruent compared to congruent trials (i.e., Stroop effect) (Stroop 1935). The RT difference between incongruent and congruent trials of about 50 ms was in line with the interference score reported in a previous study that used the same CST (mean interference score of 75 ms in Wolff et al., 2016). The interference score was larger for higher RTs (i.e., larger RT quantiles) (Pratte et al. 2010). There was a reduced interference score after incongruent trials compared to after congruent trials, that is, a conflict adaption or Gratton effect (Gratton et al. 1992). There was a training effect in that participants’ overall responses were faster the second time they performed the task (Maylor and Rabbitt, 1987; see supplement). This training effect was more pronounced for incongruent trials (see supplement). This result is in line with previous observations that training decreases interference scores in the Stroop task (Chen et al., 2013). Task difficulty was similar (i.e., ER of about 6% across congruent and incongruent trials) compared to that reported in previous studies using the same CST (Wolff et al. 2016) or the Color Stroop task (Marinkovic et al. 2012).

Clamped moderate alcohol exposure did not affect attentional inhibition, as measured with the RT interference score in the CST. We also found no alcohol × trial-type interaction for ER and IES. That is, the lack of an effect of acute alcohol exposure on RT interference was not due to a speed–accuracy trade-off. These results are consistent with recent studies using Stroop interference scores to measure attentional inhibition (Marinkovic et al. 2012; Bartholow et al. 2018). Notably, alcohol was ingested in these studies and thus the resultant exposures were not as consistent across subjects. Our supplemental analyses did not yield any specific effect of moderate alcohol exposure on attentional inhibition, that is, an effect on RT interference scores at certain RT quantiles or an effect on conflict adaptation. There was also no effect of alcohol exposure on the RT interference score attributable to the four response buttons. Taken together, our findings support the conclusion of no effect of acute alcohol on attentional inhibition, as measured with Stroop interference scores, under a moderate and steady aBAC.

The current study did not directly compare performance on the CST to the SST and the Go/NoGo task. However, our results fit well with a rich literature suggesting that acute alcohol exposure spares attentional inhibition, while response inhibition is impaired (de Wit et al. 2000; Marczinski et al. 2005; Loeber and Duka 2009; Marinkovic et al. 2012; Gan et al. 2014; Korucuoglu et al. 2015; Stock et al. 2016). Such a selective effect of acute alcohol on behavior was observed when response and attentional inhibition were examined in the same sample (Bartholow et al. 2018). As of yet, no study has directly compared the neurobiological effects of acute alcohol on response inhibition and attentional inhibition. Separate neuroimaging studies demonstrated a decrease in task-induced neuronal activity in the anterior cingulate cortex by acute alcohol exposure for the SST (Schuckit et al. 2012), Go/NoGo task (Anderson et al. 2011) and the Stroop task (Marinkovic et al. 2012). Critically, the activated brain regions and regions affected by alcohol did not completely overlap for the three tasks (for a comprehensive overview of the neural basis of response inhibition, see Chambers et al., 2009). Notably, studies that use a manipulation other than alcohol also show selective effects on response inhibition at the behavioral level (e.g., methylphenidate as in Scheres et al. 2003; transcranial magnetic stimulation as in Chambers et al. 2007).

Findings on the selective behavioral and neurobiological effects of acute alcohol exposure and other manipulations on inhibition are consistent with the view that response and attentional inhibition are distinct entities (Nigg 2000; Wignall and de Wit 2011; Morooka et al. 2012; Diamond 2013; Khng and Lee 2014; Bender et al. 2016; Tiego et al. 2018). First, the differences are apparent at the level of the associated tasks, each involving a different motivation. The Stroop task requires suppressing attention to a distracting stimulus (prepotent response) and directing attention towards another response (attentional inhibition). The SST and Go/NoGo tasks require the suppression of a prepotent response in order to not respond at all instead (response inhibition) (Diamond 2013). Furthermore, in a Stroop task participants do not necessarily intend to use the prepotent response first, while in the SST and Go/NoGo task the prepotent response is the one that is intended (Nigg 2000; Wignall and de Wit 2011). Second, a number of behavioral studies that examined both facets of inhibition in the same participants and did not use an alcohol manipulation support the assumption of different neurocognitive mechanisms. For example, correlations between task performance in the SST and Go/NoGo task, on the one hand, and the Stroop task, on the other, are only low to moderate (Miyake et al. 2000; Friedman et al. 2008; Khng and Lee 2014; Wolff et al. 2016; Bartholow et al. 2018). In addition, it has been shown with structural equation modeling that the SST and Go/NoGo task, on the one hand, and the Stroop task, on the other, form separate latent variables describing different facets of inhibition (Tiego et al. 2018).

Besides the presumed difference in the neural basis of response and attentional inhibition, there is another complementary explanation for the lack of an alcohol effect on Stroop interference: Participants may adopt an automated behavior to complete the CST. An automated behavior, in turn, is relatively unaffected by even high alcohol exposures (Beaton et al. 2018; Zink et al. 2019). More specifically, participants may have focused on constantly “counting” the number of digits after learning that the prepotent response (i.e., “reading” the numerals) is counterproductive. A transition to such an automated behavior was favored by our task design in that the CST did not include a switch or neutral condition which could have indicated some benefit of choosing the prepotent response. As a result, the prepotent response (i.e., “reading”) interfered less with the task-relevant stimulus (i.e., “counting” the number of digits). Such an automated behavior still clearly requires inhibition in incongruent trials as shown by a robust main effect of trial-type. However, resisting a strongly unintended and unfavorable prepotent response is likely a process that demands a lower amount of inhibition than resisting the prepotent response if it were to some extent useful and therefore intended. Notably, it has also been shown for the SST that different participants use different behaviors (i.e., response strategies) that are affected differently by acute alcohol exposure (Plawecki et al. 2018).

Another key finding of our analyses was that alcohol exposure resulted in an increase in RTs regardless of trial-type. This increase was within the range that was found previously (Marinkovic et al. 2012; Bartholow et al. 2018). Critically, we do not attribute the general response slowing to an impairment of the motor response because mean RTs in SST-“Go-trials” were not affected by a similar aBAC clamp in a previous study (Gan et al. 2014). Therefore, the alcohol-associated general response slowing may be explained through impairment in “counting,” an approach that may be used by participants in both congruent and incongruent trials. Similarly, slowing may be partially explained by a reduction in processing speed under alcohol exposure (Tzambazis 2000; Fillmore et al. 2009). The alcohol-associated general response slowing could also explain the decrement in performance in studies that used the Stroop task with only incongruent stimuli and reported an effect of alcohol on attentional inhibition (Rose and Duka 2007, 2008). Taken together, these results clearly emphasize the negative impact of alcohol on cognitive performance by a general increase in RT and IES in the Stroop task. However, attentional inhibition was not impaired, which brings about conceptual clarity to the impact of acute alcohol exposure.

The clinical significance of our results can be assessed only in the context of previous clinical studies. Response inhibition is more reliably impaired than attentional inhibition in those with alcohol use disorder (Wilcox et al. 2014). Additionally, it has been shown that response inhibition ability, but not attentional inhibition ability, predicted relapse and drop-out from treatment in patients with alcohol use disorder (Rupp et al. 2016; Tilden et al. 2018; Barreno et al. 2019; van Emmerik-van Oortmerssen et al. 2020). Other studies demonstrated that not only relapse, but also treatment response could be predicted using response inhibition ability (Czapla et al. 2016). Furthermore, successful training of response inhibition ability has been shown to reduce excessive alcohol use (Houben et al. 2011). Considering both past clinical studies and our current findings, it is conceivable that attentional inhibition may not be as relevant as response inhibition to the development and persistence of alcohol use disorder as well as relapse. We do not exclude that low attentional inhibition is a risk factor for developing alcohol use disorder but it may not be part of the vicious cycle that sustains the disorder. Instead, it might be a neurocognitive resource in patients that may be utilized in treatment. For example, well-functioning attentional inhibition may reduce elevated alcohol use that results from heightened attentional bias to alcohol (Roberts et al. 2014; Basanovic et al. 2017).

The interpretation of the results of the current study must take five specific features of our Stroop task and the participant sample into account. First, in contrast to previous studies, we used the CST instead of the Color Stroop task. In the CST, there is a natural mapping between stimulus and response, which we consider a benefit as this feature reduces the involvement of additional cognitive processes. Although RT interference scores for the CST (i.e., 50–75 ms) were lower compared to the Color Stroop task (i.e., 130–150 ms in Bartholow et al., 2018; Marinkovic et al., 2012), we do not expect a lower sensitivity for measuring attentional inhibition and its modulation by alcohol in our study. We attribute higher interference scores for the Color Stroop task to an overall slower task performance rather than to increased sensitivity. Consistent with this interpretation, we observed higher RT interference scores for higher RT quantiles (Fig. 3) but no effect of alcohol with respect to any specific RT quantile.

Second, neutral or switch conditions were not included in our task, primarily to make results comparable to those of a previous study (Wolff et al. 2016). It is possible that the CST would yield an alcohol-associated increase in interference scores if switch conditions, and therefore a balance between control and flexibility were required. Deficits in shifting ability after acute alcohol ingestion have been demonstrated in pure switching paradigms, with the alcohol effect being less pronounced with higher pre-drink performance (Korucuoglu et al. 2017) or with a lower degree of memory required in the shifting task (Wolff et al. 2018). However, Marinkovic et al. (2012) found no significant alcohol × trial-type interaction, even though two switch conditions were included in their modified Color Stroop paradigm. The assessment of switch costs would have also provided more insight into the mechanisms underlying the lack of an effect of alcohol on attentional inhibition. For example, switch costs would have been higher under alcohol exposure when relying more strongly on “counting” in an interference condition.

Third, we did not include practice blocks to ensure a sufficient error rate (≥ 10%) in order to examine specific effects of acute alcohol intake on post-error adjustment of attentional inhibition (see Bailey et al. 2014).

Fourth, the current participant sample had a higher proportion of male participants (90% versus 50–75%) and higher mean age (about 6 years) than those of comparable studies. However, sex differences in Stroop interference scores are minor (MacLeod 1991; Van der Elst et al. 2006), and so are increases in interference scores observed over 6 years of aging (e.g., Figs. 1 and 2 in Van der Elst et al., 2006). Moreover, mean interference scores in this study were similar to those of a previous study using the exact same task in a younger and more heterogeneous sample (Wolff et al. 2016). However, it is possible that even weight-adjusted doses of alcohol may result in differential exposures and thus produce a sex effect (Frezza et al. 1990; Mumenthaler et al. 1999). This circumstance might potentially lead to larger or different alcohol-induced changes or impairments in attentional inhibition in females, as compared to males (Fillmore and Weafer 2004b; Weafer and Fillmore 2012) if exposure properties are not carefully considered. In contrast, and as might be expected given the variability in alcohol exposures after oral consumption, another study found no difference in the effects of acute alcohol on Stroop performance in females, as compared to males (Marinkovic et al. 2012). Consequently, our sex balance may be considered a weakness but our administration method an advantage for the detection of such effects.

Fifth, our sample was recruited for medium-to-high risk drinking behavior versus other patterns such as binge drinking (Stock et al. 2016) or social drinking (Marczinski et al. 2005; Gan et al. 2014; Roberts et al. 2016). It could be argued that our participants developed tolerance and were less sensitive to an alcohol exposure. For example, acute effects of alcohol on response inhibition could not be fully replicated in a sample of heavy drinkers in a recent study (Baines et al. 2019). However, the general response slowing by acute alcohol exposure in the current study was within the range of those reported previously (Marinkovic et al. 2012; Bartholow et al. 2018). Furthermore, participants in our study reported moderate intoxication under an aBAC clamp of 80 mg%. These observations suggest no significant tolerance to acute alcohol exposure in our sample. We also consider chronic effects of alcohol on cognition to be insignificant in our sample: mean interference scores in the CST were similar to a previous sample of lower risk participants (Wolff et al. 2016); the educational level of the participant sample was above average, indicating high levels of cognitive performance; none of the participants had an alcohol use disorder.

In addition to these five specific features, further limitations are: (a) the lack of an SST or a Go/NoGo task for a direct comparison of response and attentional inhibition, (b) the lack of a Stroop task with alcohol-related stimuli or other assessments of attentional bias to alcohol-related stimuli, (c) no assessment of effects of alcohol on neurocognitive domains that are closely related to Stroop performance (e.g., processing speed and working memory; Diamond, 2013; Tzambazis, 2000) and (d) no concurrent neuroimaging during the task. Larger future studies designed to address these limitations promise further insights into the neuronal mechanisms underlying inhibition.

Nevertheless, our findings advance understanding of the effects of acute alcohol on inhibition and fill a gap in the literature. Although we found a general increase in Stroop response times induced by acute moderate and constant alcohol exposure, there was no impairment of attentional inhibition as measured with Stroop interference scores. There are two, not mutually exclusive explanations for the absence of alcohol exposure effects on attentional inhibition: First, different inhibition tasks measure different facets of inhibition, which in turn are affected differently by alcohol (Miyake et al. 2000; Friedman et al. 2008; Diamond 2013; Khng and Lee 2014; Wolff et al. 2016; Bartholow et al. 2018; Tiego et al. 2018). Second, participants may have adopted an automated behavior, which is less sensitive to even high alcohol exposures. Our findings are relevant to both experimental psychology and clinical psychiatry and psychotherapy as they inform complex models of inhibition and illuminate cognitive processes spared by alcohol that may be a cognitive resource to be employed in treatment.

## Supplementary Information

ESM 1 (PDF 812 kb)
