# Supplementary material for: Acute alcohol does not impair attentional inhibition as measured with Stroop interference scores but impairs Stroop performance
Source: Psychopharmacology (Berl). 2021 Mar 4;238(6):1593–607. doi: 10.1007/s00213-021-05792-0 (PMC8139883; doi:10.1007/s00213-021-05792-0)
Supplement: Supplementary file 1 — (PDF 812 kb) [file 213_2021_5792_MOESM1_ESM.docx]

SUPPLEMENT

Acute alcohol does not impair attentional inhibition as measured with Stroop interference scores but impairs Stroop performance

Psychopharmacology

P. Riedel [1, 2, 3], M. Wolff [1, 4], M. Spreer [1], J. Petzold [1, 2], M. H. Plawecki [5], T. Goschke [2, 4], U. S. Zimmermann [1, 6], M. N. Smolka [1, 2*]

[1] Department of Psychiatry and Psychotherapy, Technische Universität Dresden, Fetscherstraße 74, 01307 Dresden, Germany

[2] Neuroimaging Center, Technische Universität Dresden, Würzburger Straße 35, 01187 Dresden, Germany

[3] UCLA Semel Institute for Neuroscience & Human Behavior, David Geffen School of Medicine, 760 Westwood Plaza, Los Angeles, CA 90024, USA

[4] Department of General Psychology, Technische Universität Dresden, Zellescher Weg 17, 01069 Dresden, Germany

[5] Department of Psychiatry, Indiana University School of Medicine, 355 West 16th Street, Indianapolis, IN 46202, USA

[6] Department of Addiction Medicine and Psychotherapy, kbo-Isar-Amper-Klinikum München-Ost, Vockestraße 72, 85540 Haar, Germany

* Correspondence: Michael N. Smolka, Section of Systems Neuroscience, Technische Universität Dresden, Würzburger Straße 35, 01187 Dresden, Germany; michael.smolka@tu-dresden.de

1871 words, 5 figures, 7 tables

Supplementary Methods

The current study was part of a larger clinical research project (ClinicalTrials.gov identifier: NCT02652585, EudraCT Number: 2015-002831-16, Sponsor Protocol Number: TUD-TEMANX-065). All study procedures were approved by the Koordinierungszentrum für Klinische Studien Dresden (KKS; Coordination Center for Clinical Studies Dresden) and the Ethics Committee of the Technische Universität Dresden (TUD).

Based on a simulated naltrexone effect on the primary outcome measure of the clinical trial (https://clinicaltrials.gov/ct2/show/NCT02652585), a test significance level of $\alpha = 0.05$ and a power of 80%, a sample size of 17 participants per treatment arm (naltrexone/placebo) was computed using nQuery software (Statsols, Cork, Ireland). Assuming a drop-out rate of 8 participants per treatment arm, the sample size was set to 50. Participants were recruited via advertisements in public newspapers, advertisements on social media, and flyers. After a telephone screening (N = 819) and an on-site screening (N = 196), 46 participants consented and were eligible for the study.

The entire project was conducted on seven separate days at the University Hospital Carl Gustav Carus Dresden (initial telephone screening, screening day on site, clinical study visits 1, 2 and 5) and the Neuroimaging Center of the TUD (clinical study visits 3 and 4). The time frame from clinical study visits 1 to 5 was within 31-34 days for each participant. Participants were randomized at the beginning of the clinical trial to either receive the nonselective opioid receptor antagonist naltrexone or placebo in a randomized, double-blind design over the entire course of the study. Participants received approximately €300.00 compensation for participating in this study. The Counting Stroop task (CST) was a secondary outcome measure of the clinical trial (please refer to https://clinicaltrials.gov/ct2/show/NCT02652585 for the primary outcome measure and further details). CST data was collected from 18 February 2016 to 31 August 2017.

<u>Naltrexone Intervention</u>

After inclusion in the clinical trial, participants were randomized to either a verum group or a placebo group at their first clinical study visit (CSV). Randomization was double-blind and performed by the Koordinierungszentrum für Klinische Studien Dresden (KKS; Coordination Center for Clinical Studies Dresden) via a central block design. Subsequent to CSV 1, participants took the study medication for 28 days. If assigned to the verum arm, participants received naltrexone with a dose of 25 mg/d from day 1 to day 3 and with a dose of 50 mg/d from day 4 to day 28. Participants assigned to the placebo arm received 28 placebos from day 1 to day 28. This resulted in the between-subjects factor medication (Naltrexone/Placebo) (results will be reported in Spreer et al., in preparation). The study medication was well tolerated. Only a few adverse events (AEs) occurred and were usually mild (e.g., headache, nausea, fatigue, sleep disturbance). There was no dose adjustment as a clinical effect of less than 50 mg naltrexone per day has not been demonstrated. One serious adverse event (SAE) occurred as one participant required in-patient treatment, but this SAE was not related to the study medication or study procedures.

<u>Blinding to the Alcohol Administration</u>

Participants were instructed that alcohol would be administered in both sessions, that is, a higher amount of alcohol in one session and a lower amount in the other, with a maximum aBAC of 80 mg%. That is, participants were not informed about the normal saline condition. Participants were blinded in terms of quantity, taste and

amount of alcohol. Participants were blind to the aBAC readings as there was a short delay between exhalation and the determination as well as display of the measurement.

Assessment of Subjective Alcohol Effects

Participants completed a visual analogue scale (VAS) on subjective alcohol effects. All items of the VAS are presented in Table S1. Participants were asked to indicate to what extent each point reflected their current state (yes, extremely strong |—–| no, not at all). Only on item 6, participants reported an absolute number. Participants rated each item on the VAS in each session (Alcohol/NaCl), and at three different timepoints: [I] before the start of the intravenous infusion; [II]: I + 25 min (i.e., before the start of the Counting Stroop task); [III]: I + 75 min (i.e., after MRI).

Influence of Alcohol Administration Order on Session/Training Effects

Session/training effects have been described for the Stroop Task (Chen et al., 2013). These training effects were not of interest to the current study. However, we performed supplemental analyses to exclude any influence of alcohol administration order on training effects as a confounding mechanism. We tested whether training effects were affected by the order in which alcohol was administered (Alcohol_1st_Session/Alcohol_2nd_Session). The order was counterbalanced across the sample with about half of the participants receiving alcohol in the first session (N = 18) and about half of the participants receiving alcohol in the second session (N = 22). We averaged the mean reaction time (RT), error rate (ER) and inverse efficiency scores (IES) of each participant across the first and second session, both with regard to trial-type (Incongruent/Congruent) and regardless of trial-type (mean of incongruent and congruent trials). To examine differences in all average scores between the group of participants that received alcohol first and the group of participants that received alcohol second, we used paired sample t-tests. No significant differences in average scores between these two groups would indicate that training effects in the Counting Stroop task (CST) were not affected by the order in which alcohol was administered. It would also allow not including order as a between-subjects factor in the main analyses on effects of acute alcohol exposure on attentional inhibition.

Session/Training Effects

We performed 2 X 2 X 2 factorial mixed-design analyses of variance (MD-ANOVAs) including the between-subjects factor order (Alcohol_1st_Session/Alcohol_2nd_Session) in addition to the within-subject factors trial-type (Incongruent/Congruent) and alcohol (Alcohol/NaCl) to assess session/training effects. Within this model, the order X alcohol interaction reflected the main effect of session and the order X alcohol X trial-type interaction reflected the session X trial-type interaction. These supplemental MD-ANOVAs were performed for RT, ER, and IES scores.

Effect of Response Button

The transfer of the responses “2”, “3” and “4” to the respective single button presses could have been less intuitive than for the response “1”. For example, transitioning from response “3” to the single button “b” (or right index finger) could have slowed or accelerated responses and thereby masked effects of acute alcohol on attentional inhibition. Likewise, trained responses with the right index finger in mostly right-handed participants could have accelerated responses and thus masked effects of acute alcohol. To exclude that an effect of acute

alcohol on interference scores was masked by an effect of button-press, we additionally performed a 2 X 4 factorial repeated measures ANOVA with the within-subject factors alcohol (Alcohol/NaCl) and button-press (“Y”=1/“C”=2/“B”=3/“M”=4). The dependent variables were the interference scores for RT, ER, and IES.

Gratton Effect

To assure overall feasibility of the CST design to measure attentional inhibition during acute alcohol exposure and to capture potentially more specific effects of acute alcohol on attentional inhibition, we performed an additional 2 X 2 X 2 factorial repeated measures ANOVA with the within-subject factors alcohol, current trial-type and previous trial-type.

Alcohol Effects in Placebo Arm of the Naltrexone Intervention

Twenty-three participants received placebo instead of naltrexone. 2 X 2 factorial repeated measures ANOVAs with the within-subject factors trial-type (Incongruent/Congruent) and alcohol (Alcohol/NaCl) were additionally conducted on RTs, ERs, and IES in this subsample. Effect sizes of the main and interaction effects were compared to the full sample to support our notion that the naltrexone intervention had no relevant effect on the results presented in the current study.

Supplementary Results

Subjective Alcohol Effects

Results for subjective alcohol effects reported on a VAS are presented in Fig. 1. Participants rated the anticipated number of drinks (item 6) and the subjective feeling of being drunk (item 7) higher in the alcohol session compared to the NaCl session, but these two items were not 0 in the NaCl session. Although these results indicate that blinding to the intervention was not fully achieved, we are not aware of a more effective approach of blinding an alcohol administration. Additional expected (side) effects of alcohol hardly differed between sessions. Notably, participants reported moderate intoxication under an aBAC of 80 mg%, which suggests that the sample had not developed a strong tolerance to alcohol.

Influence of Acute Alcohol on Session/Training Effects

There were no significant differences between the group that received alcohol first and the group that received alcohol second in any of the scores (i.e., mean RT, ER, and IES scores) that were averaged across sessions. That is, there were neither differences in averaged incongruent trials, congruent trials nor combined trials (mean of incongruent and congruent trials), nor was there a difference in the averaged interference scores (all $|t| < 1.1$, all $p > 0.3$). That is, training effects (see below) were not different with regard to the order in which alcohol was administered.

Session / Training Effects

Statistical results on the CST for the full-factorial model are presented in Table S5. Estimated marginal means (EMM) for incongruent and congruent trials are presented as a function of alcohol in Fig. S2 and as a function of session in Fig. S3. Equivalent results were obtained for RTs, ERs and IES (see Table S5). ERs were generally

low ($M = 0.055$, $SD = 0.052$). Therefore, we focus only on RTs from here on. The 2 X 2 X 2 factorial MD-ANOVA yielded no significant alcohol X trial-type interaction ($F(1,38) = 0.58$, $p = 0.45$). There were significant main effects of alcohol ($F(1,38) = 14.72$, $p < 0.001$), trial-type ($F(1,38) = 204.46$, $p < 0.001$) and session ($F(1,38) = 16.75$, $p < 0.001$) as well as a significant session X trial-type interaction ($F(1,38) = 8.56$, $p = 0.006$). With regard to session/training effects, RTs decreased by 18 ms from the first to the second session. The session X trial-type interaction was driven by a higher decrease in RTs for incongruent trials (25 ms) than for congruent trials (13 ms).

Effect of Response Button

A 2 X 4 factorial repeated measures ANOVA with the within-subject factors alcohol (Alcohol/NaCl) and button-press (“Y”=1/“C”=2/“B”=3/“M”=4) showed no significant button-press X alcohol interactions on the interference scores for RT, ER, and IES (all $p > 0.7$). EMMs for RTs by button-press are presented in Fig. S4.

Gratton Effect

A 2 X 2 X 2 factorial repeated measures ANOVA with the within-subject factors alcohol, current trial-type and previous trial-type showed a significant current trial-type X previous trial-type interaction (i.e., conflict adaptation or Gratton effect) ($F(1,39) = 47.83$, $p < 0.001$). The Gratton effect was not affected by alcohol, that is, there was no significant alcohol X current trial-type X previous trial-type interaction ($F(1,39) = 0.10$, $p = 0.754$). An overview of all results of the supplemental statistical analysis on the Gratton effect is presented in Table S6 and Fig. S5.

Alcohol Effects in Placebo Arm of the Naltrexone Intervention

Table S7 shows the effect sizes for the main and interaction effects in the full sample compared to the subsample that was assigned to the placebo arm of the naltrexone intervention. As effect sizes did not substantially differ between the full sample and the subsample, a relevant effect of the naltrexone intervention on the results presented in the current study is unlikely.

Supplementary Figures

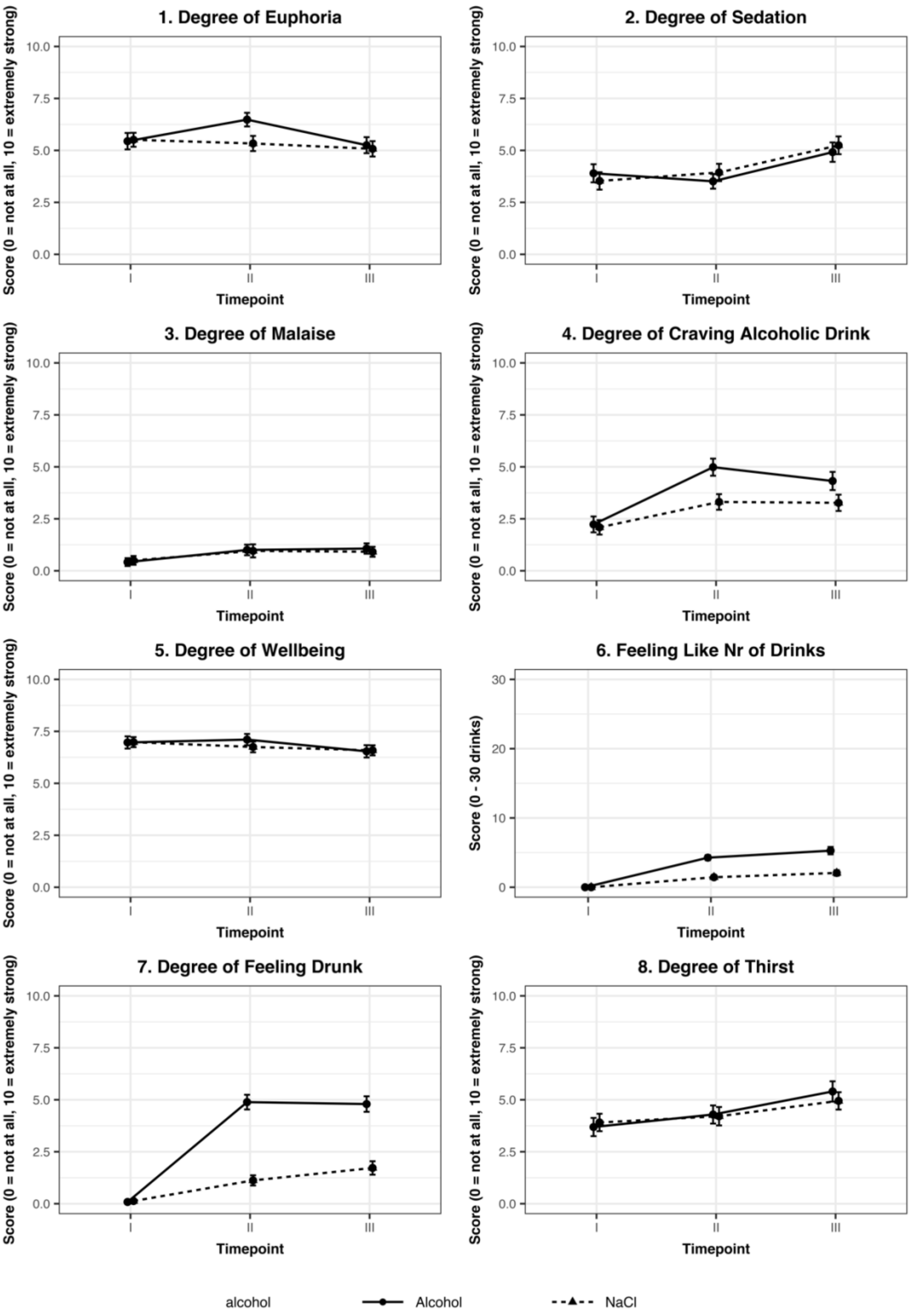

Fig S1 *Descriptive statistics Visual Analogue Scale (VAS)*. Participants rated each item on a VAS in each session (Alcohol/NaCl) at three different timepoints (I: before the start of the intravenous infusion; II: I + 25 min, i.e., before the start of the Counting Stroop task; III: I + 75 min). Presented are mean ± one standard error.

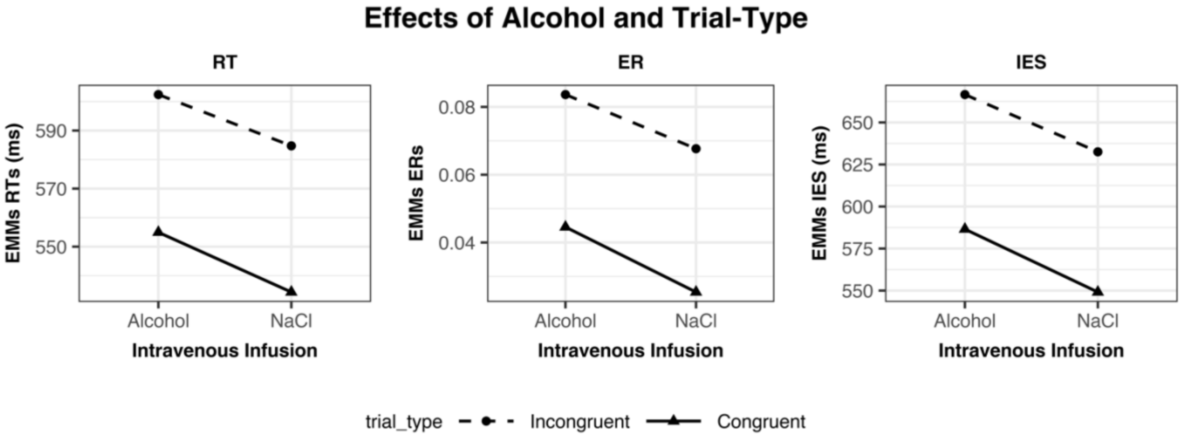

Fig S2 *Effects of alcohol and trial-type on RTs, ERs and IES.* Estimated marginal means (EMM) of RTs, ERs and IESs for each trial-type are shown as a function of alcohol. Interference scores are defined as the difference between incongruent and congruent trials. For an overview of the results of the statistical analyses please refer to Table S5. *RT* reaction time, *ER* error rate, *IES* inverse efficiency score

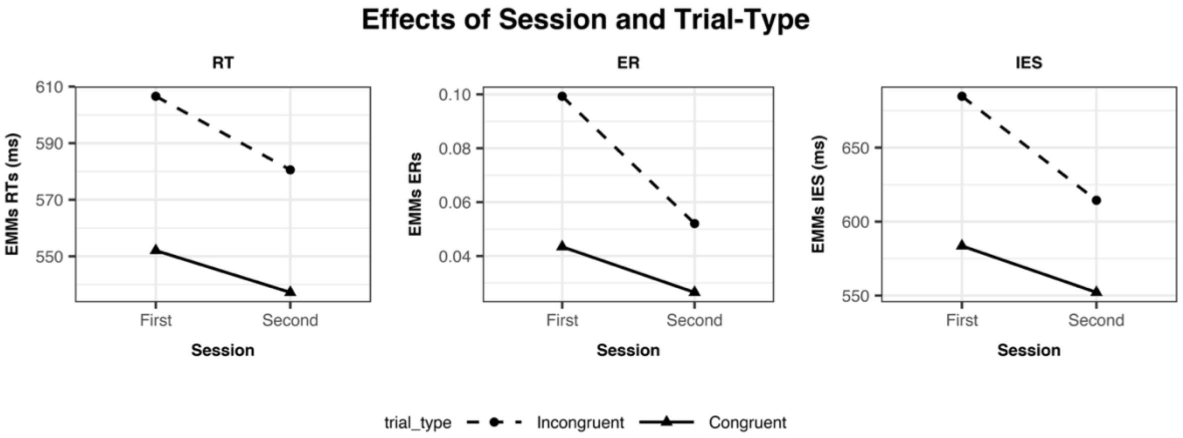

Fig S3 *Effects of session and trial-type on RTs, ERs and IES.* Estimated marginal means (EMM) of RTs, ERs and IESs for each trial-type are shown as a function of session. Interference scores are defined as the difference between incongruent and congruent trials. For an overview of the results of the statistical analyses please refer to Table S5. *RT* reaction time, *ER* error rate, *IES* inverse efficiency score

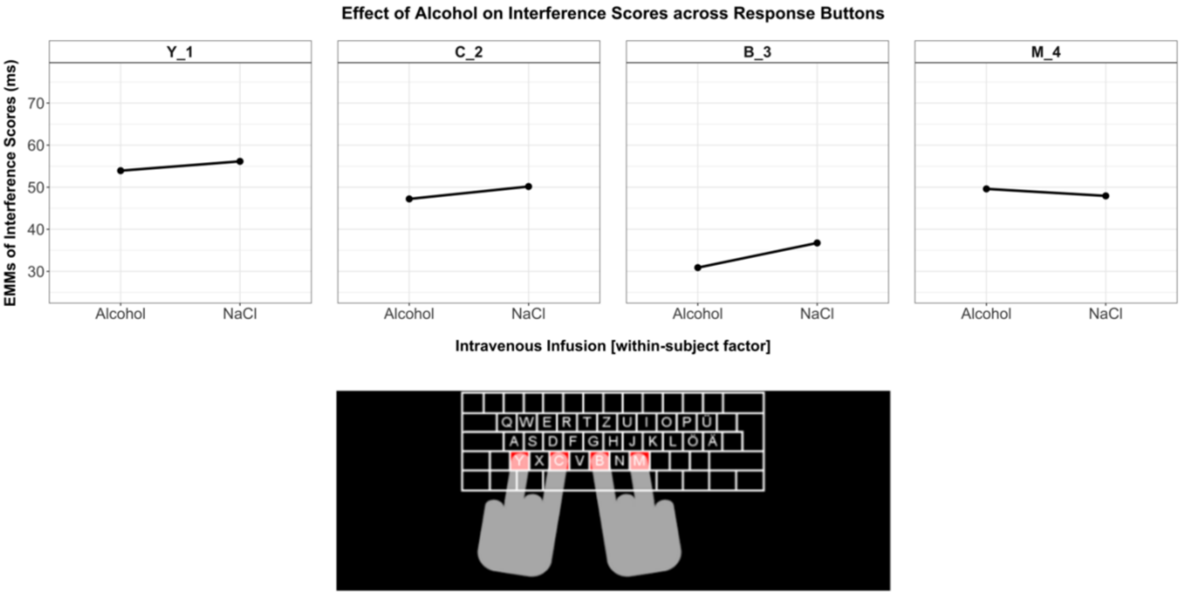

Fig S4 *Results by button-press*. There were four response keys that were naturally mapped from left to right on the keyboard (QWERTZ layout) in increasing numbers ("Y" = 1, "C" = 2, "B" = 3, and "M" = 4). Estimated marginal means (EMM) of interference scores are shown as a function of alcohol (Alcohol/NaCl) and button-press ("Y"=1/"C"=2/"B"=3/"M"=4) for reaction times (RT).

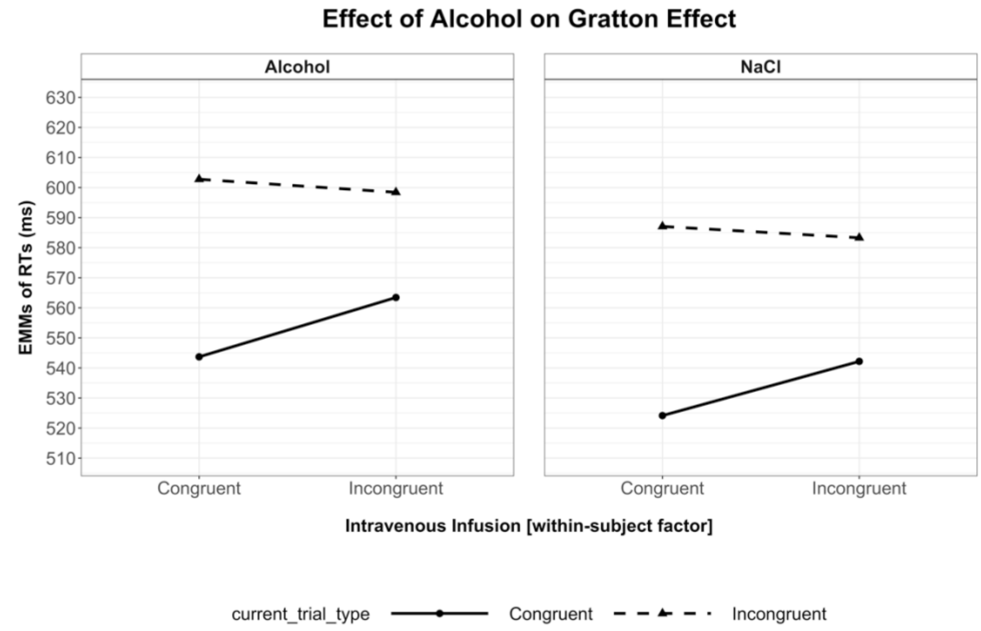

Fig S5 *Effect of alcohol on the Gratton effect*. Estimated marginal means (EMM) for reaction times (RTs) in congruent and incongruent trials are shown as a function of preceding trial-type (Incongruent/Congruent), separately for alcohol and NaCl. The Gratton effect is a reduced interference score after incongruent trials compared to after congruent trials. The Gratton effect was observed for both alcohol and NaCl.

Supplementary Tables

Table S1 *Items of a Visual Analogue Scale (VAS)*. Each item is presented in German language with English translation.

German	English
1. Ich fühle mich im Moment aufgedreht (z.B. gut gelaunt, lebhaft, energiegeladen, voll Tatendrang ...).	1. I feel exhilarated at the moment (e.g. in a good mood, lively, energetic, full of energy ...).
2. Ich fühle mich im Moment gedämpft (z.B. entspannt, ermüdet, träge ...).	2. I feel subdued at the moment (e.g. relaxed, tired, sluggish ...).
3. Ich fühle mich gerade unwohl (z.B. Übelkeit, Schwindel, Ohrensausen ...).	3. I feel unwell at the moment (e.g. nausea, dizziness, ringing in the ears ...).
4. Ich habe jetzt Lust auf noch mehr Alkohol.	4. I now feel like drinking more alcohol.
5. Ich fühle mich gerade gut.	5. I feel good right now.
6. Ich fühle mich gerade, als hätte ich ... Getränke getrunken. (0-30)	6. I feel as if I just had ... drinks. (0-30)
7. Im Moment gerade fühle ich mich betrunken	7. At the moment I feel drunk.
8. Ich habe gerade Durst (z.B. auf ein Glas Wasser, Limonade, Cola ...).	8. I am thirsty right now (e.g. longing for a glass of water, soda, coke ...).

Table S2 *Descriptive statistics for reaction times (RT): Mean, median, standard deviation (SD), maximum (Max), and minimum (Min) for total RTs are presented by trial-type (Incongruent(IC)/Congruent(C)) and alcohol (Alcohol/NaCl). In addition, descriptive statistics are presented for RT interference scores (IF) per alcohol condition.*

Descriptive Statistics	RT (ms)					
	Alc - IC	Alc - C	Alc - IF	NaCl - IC	NaCl - C	NaCl - IF
**Mean**	601	554	47	586	535	51
**Median**	595	544	48	576	544	48
**SD**	61	51	27	57	52	23
**Max**	741	669	111	713	644	101
**Min**	454	442	-6	445	424	11

Table S3 *Descriptive statistics for error rates (ER): Mean, median, standard deviation (SD), maximum (max), and minimum (min) for total ERs are presented by trial-type (Incongruent(IC)/Congruent(C)) and alcohol (Alcohol/NaCl). In addition, descriptive statistics are presented for ER interference scores (IF) per alcohol condition.*

Descriptive Statistics	ER					
	Alc - IC	Alc - C	Alc - IF	NaCl - IC	NaCl - C	NaCl - IF
**Mean**	0.08	0.04	0.04	0.07	0.03	0.04
**Median**	0.05	0.03	0.03	0.05	0.01	0.03
**SD**	0.09	0.08	0.06	0.07	0.03	0.05
**Max**	0.55	0.53	0.23	0.30	0.11	0.20
**Min**	0.00	0.00	-0.05	0.00	0.00	-0.04

Table S4 *Descriptive statistics for inverse efficiency scores (IES): Mean, median, standard deviation (SD), maximum (max), and minimum (min) for total IES are presented by trial-type (Incongruent(IC)/Congruent(C)) and alcohol (Alcohol/NaCl). In addition, descriptive statistics are presented for IES interference scores (IF) per alcohol condition.*

Descriptive Statistics	IES (ms)					
	Alc - IC	Alc - C	Alc - IF	NaCl - IC	NaCl - C	NaCl - IF
**Mean**	663	585	77	636	551	85
**Median**	634	570	71	616	532	70
**SD**	107	83	67	101	64	61
**Max**	1009	970	334	972	726	319
**Min**	477	459	12	451	430	9

Table S5 *Training effects: Main and interaction effects of 2 X 2 X 2 factorial MD-ANOVA for reaction times (RT; left), error rates (ER; middle) and inverse efficiency scores (IES; right).* The order (Alcohol_1st_Session/Alcohol_2nd_Session) X alcohol interaction reflected the main effect of session (training effect). The order X alcohol X trial-type interaction reflected the session X trial-type interaction (training effect with regard to trial-type). Values rounded to two decimals. *DFn* degrees of freedom in the numerator, *DFd* degrees of freedom in the denominator, * significant, η^2_G generalized eta-squared.

Effects	DFn	DFd	RT				ER				IES			
			F	p		η^2_G	F	p		η^2_G	F	p		η^2_G
**(Intercept)**	1	38	4941.05	< 0.01	*	0.99	44.89	< 0.01	*	0.4	2765.54	< 0.01	*	0.98
**Order**	1	38	0.86	0.36		0.02	0.77	0.39		0.01	0	0.99		< 0.01
**Alcohol**	1	38	14.72	< 0.01	*	0.03	2.13	0.15		0.02	9.15	< 0.01	*	0.04
**Trial-type**	1	38	204.46	< 0.01	*	0.17	36.75	< 0.01	*	0.08	95.66	< 0.01	*	0.19
**Session**	1	38	16.75	< 0.01	*	0.04	7.08	0.01	*	0.05	18.55	< 0.01	*	0.08
**Alcohol X Trial-type**	1	38	0.58	0.45		< 0.01	0.09	0.76		< 0.01	0.11	0.74		< 0.01
**Session X Trial-type**	1	38	8.56	< 0.01	*	< 0.01	8.29	< 0.01	*	0.01	15.17	< 0.01	*	0.01

Table S6 *Gratton effect: Main and interaction effects of the 2 X 2 X 2 repeated measures ANOVA for response times (RT).* Values rounded to two decimals. *DFn* degrees of freedom in the numerator, *DFd* degrees of freedom in the denominator, * significant, η^2_G generalized eta-squared.

Effects	DFn	DFd	F	p		η^2_G
**(Intercept)**	1	39	5001.55	< 0.001	*	0.99
**Alcohol**	1	39	9.445	0.004	*	0.03
**Current Trial-type**	1	39	237.484	< 0.001	*	0.16
**Previous Trial-type**	1	39	11.534	0.002	*	< 0.01
**Alcohol X Current Trial-type**	1	39	1.703	0.2		< 0.01
**Alcohol X Previous Trial-type**	1	39	0.031	0.861		< 0.01
**Current Trial-type X Previous Trial-type**	1	39	47.826	< 0.001	*	0.01
**Alcohol X Current Trial-type X Previous Trial-type**	1	39	0.1	0.754		< 0.01

Table S7 *Effect size for main and interaction effects of 2 X 2 factorial repeated measures ANOVA for reaction times (RT; left), error rates (ER; middle) and inverse efficiency scores (IES; right) in the total sample (ALL; N = 40; DFn = 1, DFd = 39) compared to the subsample receiving placebo (PLC; N =23; DFn = 1, DFd = 22).* Values rounded to two decimals. *DFn* degrees of freedom in the numerator, *DFd* degrees of freedom in the denominator, η^2_G generalized eta-squared.

Effects	RT		ER		IES	
	η^2_G PLC	η^2_G ALL	η^2_G PLC	η^2_G ALL	η^2_G PLC	η^2_G ALL
**(Intercept)**	0.99	0.99	0.33	0.38	0.98	0.98
**Alcohol**	0.02	0.02	0.02	0.01	0.04	0.03
**Trial-type**	0.16	0.16	0.04	0.07	0.13	0.17
**Alcohol X Trial-type**	< 0.01	< 0.01	< 0.01	< 0.01	< 0.01	< 0.01
